# The Stringent Response of *Staphylococcus aureus* and Its Impact on Survival after Phagocytosis through the Induction of Intracellular PSMs Expression

**DOI:** 10.1371/journal.ppat.1003016

**Published:** 2012-11-29

**Authors:** Tobias Geiger, Patrice Francois, Manuel Liebeke, Martin Fraunholz, Christiane Goerke, Bernhard Krismer, Jacques Schrenzel, Michael Lalk, Christiane Wolz

**Affiliations:** 1 Interfaculty Institute of Microbiology and Infection Medicine, University of Tübingen, Tübingen, Germany; 2 Genomic Research Laboratory, Infectious Diseases Service, Geneva University Hospitals and the University of Geneva, Geneva, Switzerland; 3 Institute of Pharmaceutical Biology, Ernst-Moritz-Arndt University of Greifswald, Greifswald, Germany; 4 Department of Microbiology, Biocenter, University of Würzburg, Würzburg, Germany; National Institute of Allergy and Infectious Diseases, National Institutes of Health, United States of America

## Abstract

The stringent response is initiated by rapid (p)ppGpp synthesis, which leads to a profound reprogramming of gene expression in most bacteria. The stringent phenotype seems to be species specific and may be mediated by fundamentally different molecular mechanisms. In *Staphylococcus aureus*, (p)ppGpp synthesis upon amino acid deprivation is achieved through the synthase domain of the bifunctional enzyme RSH (RelA/SpoT homolog). In several firmicutes, a direct link between stringent response and the CodY regulon was proposed. Wild-type strain HG001, *rsh_Syn_*, *codY* and *rsh_Syn_*, *codY* double mutants were analyzed by transcriptome analysis to delineate different consequences of RSH-dependent (p)ppGpp synthesis after induction of the stringent response by amino-acid deprivation. Under these conditions genes coding for major components of the protein synthesis machinery and nucleotide metabolism were down-regulated only in *rsh* positive strains. Genes which became activated upon (p)ppGpp induction are mostly regulated indirectly via de-repression of the GTP-responsive repressor CodY. Only seven genes, including those coding for the cytotoxic phenol-soluble modulins (PSMs), were found to be up-regulated via RSH independently of CodY. qtRT-PCR analyses of hallmark genes of the stringent response indicate that an RSH activating stringent condition is induced after uptake of *S. aureus* in human polymorphonuclear neutrophils (PMNs). The RSH activity in turn is crucial for intracellular expression of *psms*. Accordingly, *rsh_Syn_* and *rsh_Syn_*, *codY* mutants were less able to survive after phagocytosis similar to *psm* mutants. Intraphagosomal induction of *psmα1-4* and/or *psmβ1,2* could complement the survival of the *rsh_Syn_* mutant. Thus, an active RSH synthase is required for intracellular *psm* expression which contributes to survival after phagocytosis.

## Author Summary

The stringent response is a bacterial response to a multitude of different environmental stress conditions which is characterized by the synthesis of the messenger molecules (p)ppGpp. There is now growing evidence that these molecules also play a key role for pathogens to switch between specific phenotypic states within the host. This seems crucial for the adaptation to different microenvironments encountered during infection for instance after uptake by phagocytes. Killing of phagocytes as well as survival within these cells was proposed as major mechanisms for the success of the human pathogen *Staphylococcus aureus* to spread within the body. In the current study we demonstrate the effect of the stringent response on global gene expression in *S. aureus* and its impact on intracellular survival in human neutrophils. We reveal that a stringent response is induced after uptake of *S. aureus* in neutrophils and RSH activity is crucial for intracellular induction of *psm* expression, coding for cytotoxic phenol-soluble modulins (PSMs). Finally we show that this in turn mediates bacterial survival and escape after phagocytosis. These findings contribute to the understanding of how and where PSMs can act as potent cytolytic molecules and emphasise the importance of (p)ppGpp as an intracellular signalling molecule.

## Introduction

In most bacteria, nutrient limitations provoke the so-called stringent response, which is initiated by the rapid synthesis of the alarmones pppGpp and/or ppGpp, here referred to as (p)ppGpp. Under stringent conditions, (p)ppGpp results in the shut-down of proliferation-related activities, including the transcriptional repression of genes coding for major components of the protein synthesis apparatus (rRNA, ribosomal proteins and translation factors) as well as the inhibition of replication [1], [2], [3]. Typically genes that are presumed to be important for maintenance and stress-defence are activated under stringent conditions. However, the stringent phenotype resulting from (p)ppGpp synthesis seems to be bacteria species specific and may be mediated by fundamentally different molecular mechanisms [3].

The molecular mechanisms leading to the profound reprogramming of the bacterial cellular machinery under stringent conditions were mostly studied in *Escherichia coli*. Here, (p)ppGpp can be synthesized by either one of the two homologous enzymes: RelA and SpoT. The RelA-synthase is activated by sensing uncharged tRNAs that are bound to the ribosome. SpoT is a bifunctional enzyme that not only produces (p)ppGpp in response to diverse signals but also contains a (p)ppGpp hydrolase domain important for (p)ppGpp turnover. In *E. coli*, (p)ppGpp binds, with the help of the DksA protein, directly to the RNA polymerase (RNAP). However, even in this model organism, there is still much debate concerning how (p)ppGpp eventually leads to different promoter activities, how (p)ppGpp influences the stability of open complex formation at the initial phase of transcription and which of the promoters are indirectly regulated via secondary regulatory circuits such as alternative sigma factors or other transcription factors [1].

In other organisms, such as the gram-positive *Bacillus subtilis*, (p)ppGpp probably does not interact with the RNAP, and no DksA homologue is present. Here (p)ppGpp has been proposed to affect promoter activities only indirectly via changes of the intracellular nucleotide pool [4], [5], [6]. In this model, the nature of the initiation nucleotide (iNTP) determines whether genes are under positive or negative stringent response [4], [5], [7], [8]. (p)ppGpp synthesis is usually accompanied by a decrease in intracellular GTP concentration. rRNA promoters in *B. subtilis* initiate with GTP and a change of this base at position +1 results in a loss of regulation by (p)ppGpp and GTP. Furthermore, GTP can act as a co-factor for the repressor CodY, and thus the lower GTP levels imposed by the stringent response result in the de-repression of CodY target genes at least in some firmicutes, e.g., *B. subtilis* [9] or *Listeria monocytogenes* [10].

In most firmicutes, three genes coding for putative (p)ppGpp synthases are present. The bifunctional RSH (RelA/SpoT homologue) enzymes are typically composed of a C-terminal sensing domain and an N-terminal enzymatic domain with hydrolase and synthase functions. RelQ and RelP (also named SAS1 and SAS2, for single small alarmone synthase) are small proteins with only a putative (p)ppGpp synthase domain [11], [12].

Little is known about the stringent response in the human pathogen *Staphylococcus aureus*, most likely because of the essentiality of the major (p)ppGpp synthase/hydrolase enzyme RSH [13], [14]. Previously, we have shown that, at least under nutrient rich conditions, mutants defective only in the synthase domain of RSH (*rsh_Syn_*) are not impaired in growth [13]. Furthermore, RSH is the only enzyme responsible for (p)ppGpp synthesis in response to amino acid starvation triggered by leucine/valine deprivation or mupirocin treatment. Preliminary characterisation of the *rsh_Syn_* mutant revealed that part of its phenotype mainly the regulation of genes involved in amino-acid metabolism can be explained by the (p)ppGpp induced de-repression of the CodY regulon. CodY of *S. aureus* was previously shown to be a major regulator of virulence gene expression [15], [16]. Thus, the CodY regulon seems to be an integral part of the stringent response in *S. aureus* linking metabolic circuits and virulence. However, whether, and to what extent, other direct or indirect regulatory circuits are involved in RSH mediated stringent response is unclear. A CodY independent contribution of the stringent response to virulence could, so far, not be indicated. However, for many other pathogenic bacteria, a contribution of (p)ppGpp to bacterial virulence could be shown [17]. In particular, for many intracellular bacteria, (p)ppGpp is essential to survive and replicate in diverse host cells, i.e., epithelial and endothelial cell types and professional phagocytes.

Here, we delineate three (category I–III) main consequences of RSH-dependent stringent response in *S. aureus*. (I): Similar to other organisms, a severe down-regulation of the protein synthesis machinery was observed, and thus, the inhibitory effects of (p)ppGpp on gene expression seem to be highly conserved in bacterial species. (II): In contrast, genes that are activated upon (p)ppGpp induction are highly species specific. In *S. aureus*, they are mostly regulated indirectly via the de-repression of CodY. (III): Interestingly, seven genes, including those coding for the toxic phenol-soluble modulins (PSMs), were found to be up-regulated independently of CodY. We demonstrate that a stringent response is induced after uptake of wild-type bacteria in human neutrophils. Moreover, RSH activity is required for *psmα1-4* and *psmβ1-2* expression in neutrophils which in turn promotes survival after phagocytosis.

## Results

### Experimental setup to characterize RSH-mediated stringent response in *S. aureus*

*S. aureus* is able to mount a stringent response upon amino acid starvation, which is characterized by the generation of (p)ppGpp [18], [19]. However, little is known about the impact of (p)ppGpp synthesis on global physiological processes in *S. aureus*. Mupirocin treatment, an antimicrobial agent that inhibits the bacterial isoleucyl tRNA synthetase and therefore mimics isoleucine starvation, results in profound reprogramming of gene expression [20], [21]. Parts of these effects are mediated by (p)ppGpp. However, at least some of them were also observed in an RSH-mutant, which is devoid of (p)ppGpp synthesis after mupirocin treatment [13].

**Figure 1:**
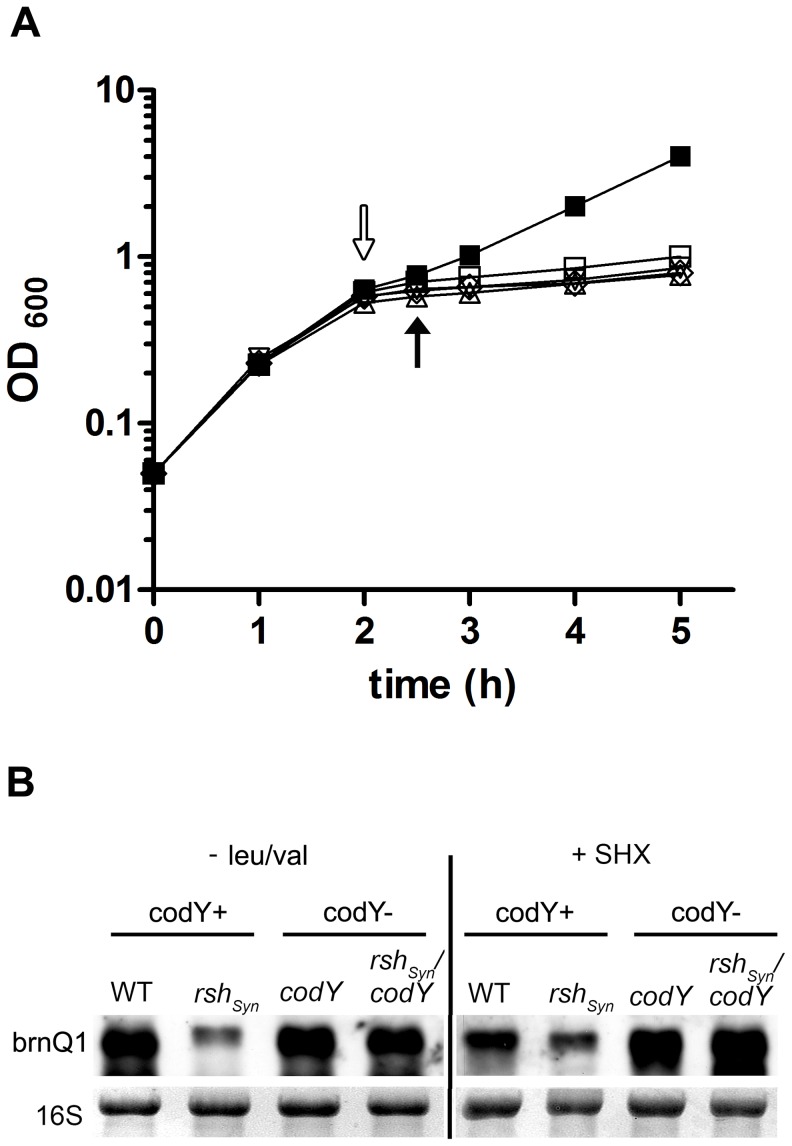
The experimental setup and the interaction of the stringent response with CodY. (A) Growth inhibition in medium lacking leucine and valine (−leu/val). *S. aureus* wild-type strain HG001 (square), *rsh_Syn_* mutant (triangle), *codY* mutant (circle) and *rsh_Syn_*, *codY* double mutant (diamond) were grown in complete CDM to an optical density of OD_600_ = 0.5 (open arrow), filtered, washed twice and re-suspended in an equal volume of CDM medium without leucine/valine (open symbols) or CDM with leucine (wild-type only, filled square). Aliquots for microarray analysis were harvested after 30 minutes (filled arrow). (B) The interaction of the two regulons CodY and stringent response under leucine/valine starvation (−leu/val) and after serine starvation induced by serine hydroxamate (+SHX) and strain HG001 (WT)), the corresponding *rsh_Syn_* mutant, *codY* mutant, and the *rsh_Sy_*_n_, *codY* double mutant were grown in CDM to the exponential growth phase (OD_600_ = 0.5) followed by further incubation in medium containing 1.5 mg/ml SHX respectively in medium without leucine/valine for 30 minutes. RNA was hybridized with digoxigenin-labelled PCR fragments. The 16S rRNA detected in the ethidium bromide-stained gels is indicated as loading control in the lower lane.

Here, we aimed to delineate the impact of (p)ppGpp synthesis induced by amino acid starvation on gene expression in *S. aureus* under defined nutrient limitation rather than after the addition of inhibitors such as mupirocin. Previously we could show that transferring *S. aureus* into a chemical defined medium (CDM) lacking the two branched-chain amino acids leucine and valine results in growth inhibition [13]. A similar experimental setup was chosen here for a further in-depth analysis including transcriptional profiling by microarray: Bacteria were grown to mid-exponential growth phase in complete CDM, filtered and shifted into a medium lacking leucine and valine (−leu/val) (Fig. 1A, open arrow). After 30 minutes of incubation in CDM −leu/val the bacteria were harvested for RNA purification (Fig. 1A, filled arrow). At this time all strains showed a slight growth inhibition compared to bacteria grown in complete CDM, which is accompanied by a clear induction of the stringent response indicated by RSH dependent (p)ppGpp synthesis and reduction of the GTP pool [13]. The *rsh_Syn_* mutant showed no accumulation of (p)ppGpp under these conditions which indicates that a contribution of the two putative (p)ppGpp synthases RelP and RelQ is negligible.

To analyze the effects mediated by (p)ppGpp synthesis we compared the transcriptional profile of wild-type strain HG001 with that of the *rsh_Syn_* mutant under the indicated stringent conditions. Previously a close link between the stringent response and the activity of the repressor CodY was demonstrated in different firmicutes [10], [13], [22], [23]. Thus, to delineate which and how many (p)ppGpp regulated genes are influenced through CodY we also analyzed the RSH mediated stringent response in *codY*-negative *S. aureus*.

In accordance to previous results [13] we could confirm by northern blot analysis that under the selected conditions of leucine/valine starvation the wild-type strain showed a significant repression of genes of the translational apparatus, such as *rpsB* (ribosomal protein S2), *infB* (translation initiation factor B) and *tsf* (translation stable factor), whereas no repression occurred in the *rsh_Syn_* mutant (data not shown). By contrast, typical CodY target genes like *brnQ1* coding for an amino acid transporter were induced in the wild-type strain (Fig. 1B). The low expression of *brnQ1* in the *rsh_Syn_* mutant however, could be restored by additional mutation of *codY*, indicating that the *rsh_Syn_* mutation affects *brnQ1* transcription through CodY (Fig. 1B). To analyze whether this observation is due to a general response to amino acid limitation, we induced serine starvation by addition of serine hydroxamate (SHX). The same transcriptional pattern was observed when compared to conditions of leucine/valine starvation (−leu/val) (Fig. 1B).

Based on the results of the northern blot hybridizations, we performed microarray analysis of wild-type *S. aureus* HG001, *rsh_Syn_*, *codY* and *rsh_Syn_*, *codY* double mutants after leucine/valine depletion (Fig. 1A). Three major categories (I–III) of stringently regulated genes were observed: I) genes that are negatively regulated by (p)ppGpp independent of CodY. II) genes that are positively influenced by (p)ppGpp through CodY de-repression and III) genes positively influenced by (p)ppGpp independent of CodY.

### Category I: Genes negatively regulated during the stringent response

**Figure 2:**
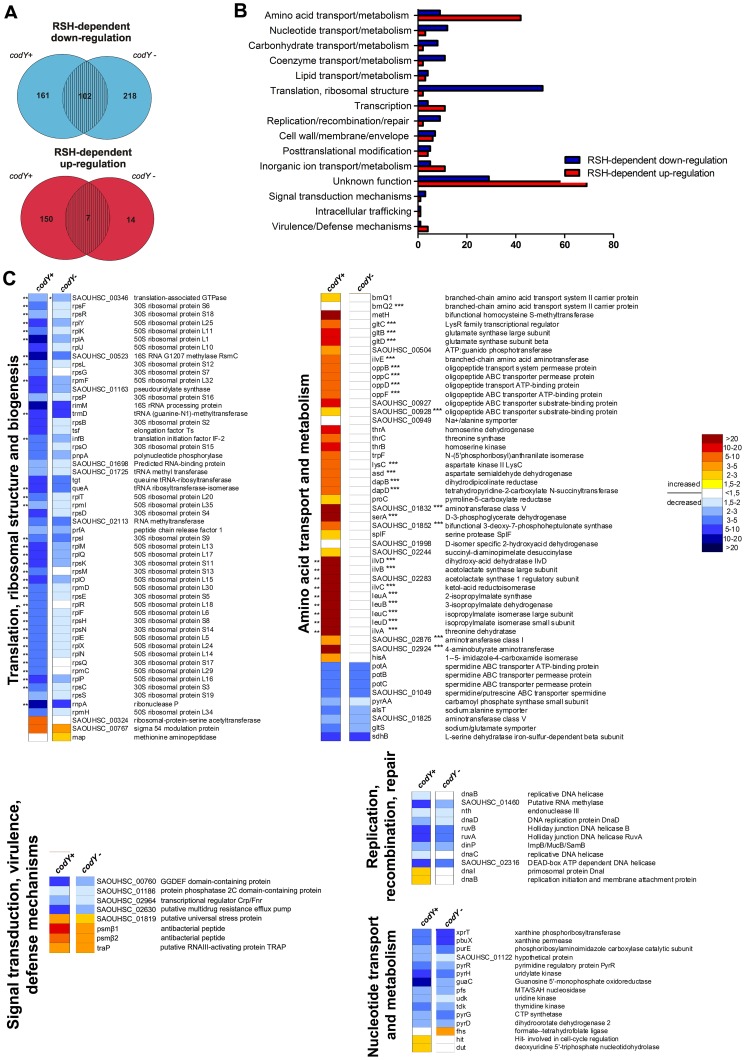
Transcriptome analysis of (p)ppGpp dependent genes during stringent response. Transcriptional changes of *S. aureus* in response to leucine/valine starvation (−leu/val) and the overlap of the two regulons CodY and stringent response. (A) Venn diagrams showing genes which are down- (blue) and up-regulated (red) during RSH mediated stringent response. A contribution of CodY to the regulation was determined by analyzing the transcriptional changes in a *codY*-positive background (WT strains vs. *rsh_Syn_* mutant) indicated as codY+ in the figure and in a *codY*-negative background (*codY* mutant vs. *rsh_Syn_*, *codY* double mutant) indicated as codY−. (B) Functional classes of genes which are down- (blue) and up-regulated (red) during RSH mediated stringent response. (C) Heat maps of gene expression ratios for indicated functional classes prominently altered in their expression. The ratios were analyzed in a *codY-*positive (codY+) and a *codY-* negative (codY−) background as described for the Venn diagrams. ** saturated signal *** genes with annotated CodY binding motifs [16].

Microarray analysis revealed that 102 genes were significantly (p-value cut-off, 0.05) down-regulated in the wild-type compared to the *rsh_Syn_* mutant in the codY positive (WT<*rsh_Syn_*) as well as in the *codY* negative background (*codY*<*rsh_Syn_*, *codY*) (Fig. 2A, overlap blue Venn diagram). This indicates that these genes are negatively regulated by (p)ppGpp independent of CodY. Since in total 161 genes were significantly down-regulated in the *codY* positive background, we also verified the 59 missing genes in the *codY* negative background. All of these genes appeared also to be down-regulated, although the difference did not reach the significance level.

Most of these CodY-independent negatively regulated genes are hallmark genes of the stringent response in other bacteria [24], [25], [26]. They are mostly involved in the translation process (Fig. 2B), such as coding for ribosomal proteins (*rps*, *rpl*), for translation initiation factor (*infB*) and translation elongation factor (*tuf*) (Fig. 2C). The rRNA processing machinery is also influenced since genes coding for ribonuclease P (*rnpA*) and *rimM* (coding for a processing protein) are repressed in an RSH-dependent manner (Fig. 2C). In addition, genes like DNA helicases (*dnaB, C, D*), part of the so called “replicon”, are negatively influenced by (p)ppGpp, and genes of the recombination/repair system (*ruvAB* and *dinP*) were also repressed under amino acid starvation due to (p)ppGpp (Fig. 2B,C). In accordance with published data from *E. coli* and *B. subtilis* [24], [25], genes of other physiological processes that are typical for dividing cells (nucleotide, lipid and coenzyme biosynthesis and transport, inorganic ion transport and protein modification) are also part of the negative stringent response in *S. aureus* (Fig. 2B,C).

In contrast to *B. subtilis* [24], *E. coli* [25], *or S. pneumoniae* [26], genes involved in cell division and cell wall biosynthesis were not found to be significantly influenced by the stringent response in *S. aureus*. However, we can not exclude that such genes are affected at later time points.

### Category II: Genes positively influenced during stringent response through interaction with CodY

We aimed to obtain a comprehensive overview of genes that are part of the predicted regulatory overlap between stringent response and CodY repression. Under stringent conditions most of the up-regulated genes were expressed only in the *codY-* positive background (Fig. 2A). An additional introduction of a *codY* mutation into this analysis (*codY* vs. *rsh_Syn_*, *codY*) abrogated the up-regulation of these genes (Fig. 2C). Thus, under amino acid deprivation many genes are de-repressed in the wild-type bacteria (WT>*rsh_Syn_*) through the relief of CodY repression, but stay constitutively repressed via CodY in the *rsh_Syn_* mutant. These genes are part of the recently described CodY regulon [15], [16] mainly involved in amino acid metabolism and transport (Fig. 2B). Accordingly, most of them possess CodY binding motifs as described elsewhere [16] and as indicated in Fig. 2C (three asterisks). It was shown previously that CodY represses also several virulence genes in part via inhibition of the quorum sensing system *agr*. Of note, none of these *codY* influenced virulence genes were found to be significantly down-regulated in the *rsh_Syn_* mutants.

### Category III: Genes positively regulated during stringent response independent of CodY

Next, we investigated if there are genes activated by (p)ppGpp with no contribution of CodY. Microarray analysis revealed that only seven genes were expressed significantly lower in the *rsh_Syn_* mutants in both, a *codY*-positive (*codY*+) and *codY*-negative background (*codY*−) (Fig. 2A, table 1). One gene coding for a putative ribosome-associated protein Y was described in *E. coli* to bind to the small ribosomal subunit and to stabilize ribosomes against dissociation when bacteria experience environmental stress [27]. Also *traP* was expressed significantly lower in the *rsh_Syn_* mutants. The function of TraP remains unknown since a proposed involvement of this protein in the activation of the *agr* system was recently disproved [28], [29], [30]. Surprisingly, two genes, part of the staphylococcal defence mechanism, coding for β1 and β2 phenol-soluble modulins (psm β1/β2) were transcribed significantly lower in both *rsh_Syn_* mutants. This result was of special interest because no other obvious virulence gene appeared to be part of the stringent response in *S. aureus*.

**Table 1:** Genes positively regulated under stringent response independent of CodY.

Annotation	Common	Description	codY+	codY−	Category
			(WT/*rsh_Syn_* 1)	(*codY/rsh_Syn_, codY* 1)	
**SAOUHSC_00401**		hypothetical protein	5.3	2.15	Function unknown
**SAOUHSC_00767**		putative ribosome-associated protein Y	7.14	4.55	Translation, ribosomal structure
**SAOUHSC_01135**	**psm β2**	antibacterial protein (phenol-soluble modulin)	8.33	3.85	Virulence/Defense mechanisms
**SAOUHSC_01136**	**psm β1**	antibacterial protein (phenol-soluble modulin)	10	4.55	Virulence/Defense mechanisms
**SAOUHSC_01819**		putative universal stress protein	3.52	2.47	General function prediced
**SAOUHSC_01907**		oxidoreductase	2.17	2.63	General function predicted
**SAOUHSC_01964**	**traP**	RNAIII-activating protein TRAP	4.76	5	General function predicted

1 : Fold changes are indicated for each comparison and displayed for genes showing statistically significant differential expression. Values corresponds to expression ratios, i.e. averaged expression levels from three independent replicate experiments (p<0.05).

### RSH-dependent induction of *psms* during the stringent response is not mediated by increased *agr* expression

**Figure 3:**
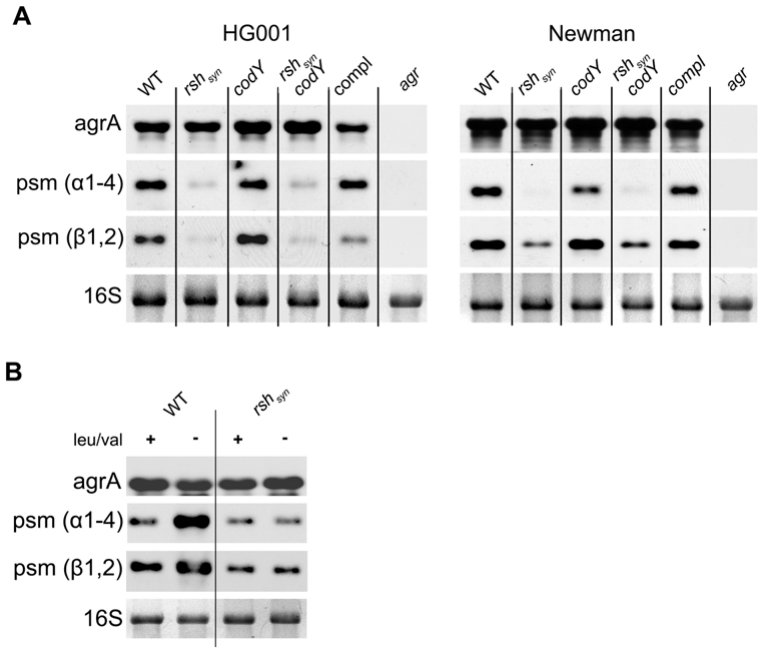
(p)ppGpp dependent *psm* expression during stringent response. (A) (p)ppGpp dependent activation of target genes in two different *S. aureus* strains. Strain HG001 (WT) and strain Newman (WT), the corresponding *rsh_Syn_* mutants, *codY* mutants, *rsh_sy_*_n_, *codY* double mutants, the complemented *rsh_Syn_* mutants (compl) and the *agr* mutants were grown in CDM to the exponential growth phase (OD_600_ = 0.5) followed by further incubation in medium without (−) leu/val for 30 minutes. (B) Wild-type strain HG001 and its isogenic *rsh_Syn_* mutant were incubated with (+) or without (−) leu/val for 30 minutes. RNA was hybridized with digoxigenin-labelled PCR fragments. The 16S rRNA detected in the ethidium bromide-stained gels is indicated as loading control in the lower lane.

The cytolytic activity of β-type PSMs was described to be minor, and their role in virulence is less pronounced compared with α-type PSMs [31]. Since no probes for α-type PSMs are present on the microarray chip, we performed northern blot hybridisation to analyze the RSH-dependent expression of these important molecules (Fig. 3). To exclude strain specific effects, *rsh_Syn_* mutants of the prototypic *S. aureus* strain Newman were also included in the analysis. After leu/val starvation, there is no or very little, α- and β-type *psm* transcription detectable in the *rsh_Syn_* mutants of strain HG001 and strain Newman (Fig. 3A) in contrast to the wild-type strain, which shows strong induction of both *psm* classes (Fig. 3B). Introducing full-length *rsh* chromosomally into the *rsh_Syn_* mutant strains could restore their deficiencies to induce *psms* expression (Fig. 3A). An involvement of CodY could be excluded since the *rsh_Syn_*, *codY* double mutant shows the same low transcription of *psms* as the *rsh_Syn_* single mutant.

Queck et al. [32] could show that *psm* expression is directly regulated via binding of the response regulator AgrA to the *psm* promoter region. Therefore, we analyzed whether *agrA* transcription is altered under the conditions tested. No significant transcriptional differences were detectable comparing wild-type strain and the corresponding mutants under leu/val deprivation (Fig. 3A/B). The response regulator AgrA also directly activates the transcription of the divergently transcribed regulatory RNAIII which in turn results in down-stream regulatory effects on several virulence genes. In the microarray analysis, no significant difference in RNAIII or other prototypic RNAIII target genes such as *hla* or *spa* were observed. To exclude possible AgrA defects, we screened the mutants for altered haemolytic activity [29]. No haemolytic deficiencies could be detected in the mutant strains (data not shown). Therefore, an AgrA defect in the *rsh_Syn_* mutants can be excluded. Nonetheless *agr* mutants exhibit no *psm* transcriptions under leu/val deprivation (Fig. 3A), supporting that *agr* activity is still essential for *psm* expression under stringent conditions. However, RSH dependent induction of *psmα1-4* and *psmβ1,2* under stringent conditions is not mediated by *agr* activation.

### RSH-dependent expression of *psms* is not mediated by the initiation nucleotide (iNTP)

**Figure 4:**
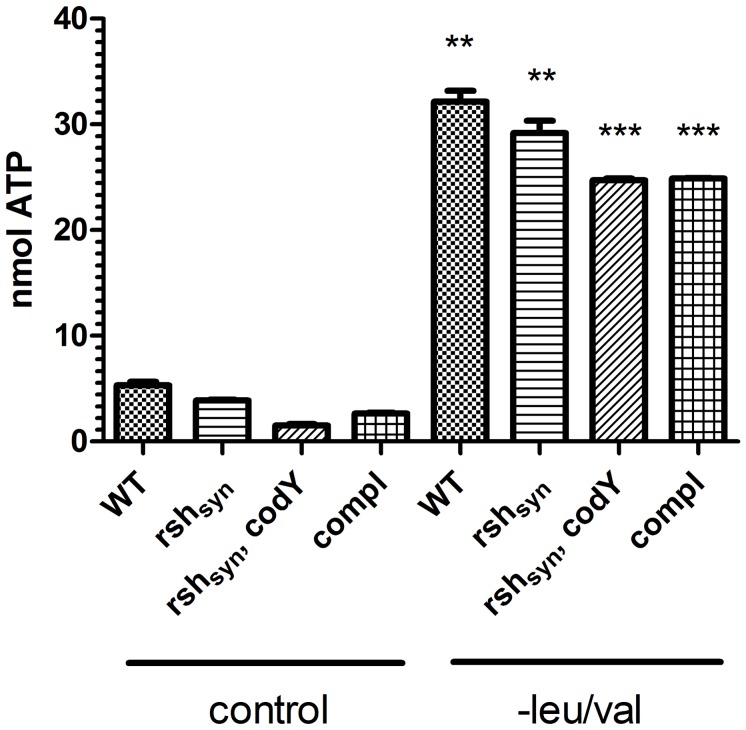
Quantitative analysis of the intracellular ATP concentrations. The intracellular ATP pools of strain HG001, the corresponding *rsh_Syn_* mutant, the *rsh_Syn_*, *codY* double mutant and the complemented *rsh_Syn_* mutant (compl) were determined by luciferase activity. The bacteria were grown in CDM to exponential growth phase (OD_600_ = 0.5) followed by further incubation in medium with (control) or without (−) leu/val for 30 minutes. The levels of significance to the corresponding control (with leu/val) were determined by the two-tailed Student t test (p<0.05).

Aside from the requirement of functional *agr*, little is known about *psm* regulation in *S. aureus*. The mechanism by which stringent response leads to increased transcription of certain genes is much under debate. In *B. subtilis*, (p)ppGpp does not appear to interact directly with RNA polymerase (RNAP) [5]. The nature of the iNTP during transcription was proposed to determine whether genes are under positive or negative stringent response. In this model, positive stringent controlled genes of *B. subtilis* are characterized by iATP, and these genes are presumably activated because of an increased ATP pool [4]. Previously, the transcriptional starting points of the *α-* and *ß-psms* were identified as an adenine ribonucleotide [32]. To analyze whether induction of phenol-soluble modulins in the wild-type strain correlates with an increased ATP pool, the intracellular ATP concentration was measured (Fig. 4). A significant increase of the intracellular ATP pool was detectable upon amino acid deprivation in all strains. However, no significant difference was found between the strains analyzed (Fig. 4). Thus, the ATP levels cannot explain the observed differences in *psm* expression between wild- type and *rsh_Syn_* mutant.

### RSH-dependent survival in polymorphonuclear leukocytes (PMNs)

**Figure 5:**
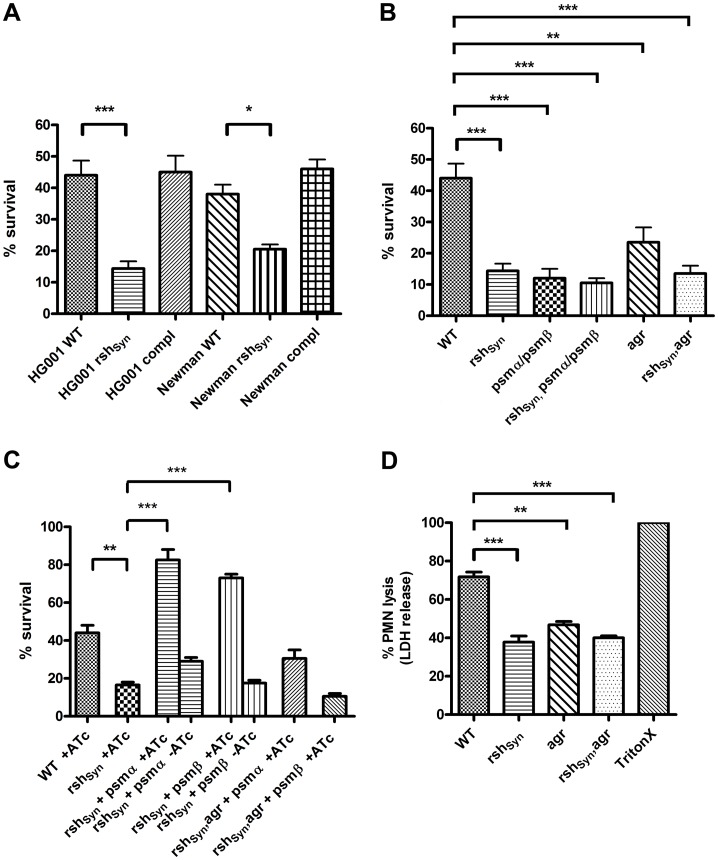
Phagocytosis killing assays and a PMN lysis assay with different *S. aureus* strains. (A) and (B) Survival of wild-type *S. aureus* strain HG001 and strain Newman (WT), and their isogenic *rsh_Syn_* mutants, complementation strains (compl), *psmα*, *psmβ* double mutants (HG001), *rsh_Syn_*, *psmα*, *psmβ* triple mutants (HG001), *agr* mutant (HG001) and *rsh_Syn_*, *agr* double mutant (HG001) were determined after phagosomal uptake by counting colony-forming units after 60 min incubation. Bacterial cells used for the experiment were harvested at similar points of growth at an OD_600_ of 1.0. The levels of significance were determined by the two-tailed Student t test (p<0.05). (C) Complementation of survival of the *rsh_Syn_* mutant with tetracycline inducible *psmα* and *psmβ*. Therefore membrane permeable anhydrotetracycline (ATc) 0.1 µg/ml was used to induce *psm* expression after phagosomal uptake of the bacteria. The levels of significance were determined by the two-tailed Student t test (p<0.05). (D) Lysis of PMNs, as measured by release of lactate dehydrogenase (LDH) activity. PMN lysis was determined after a phagocytotic interaction of 60 min with *S. aureus* strain HG001 wild-type, corresponding *rsh_Syn_* mutant, *agr* mutant and *rsh_Syn_*, *agr* double mutant. As a positive control 2% TritonX were used. The levels of significance were determined by the two-tailed Student t test (p<0.05).

Since α-type PSMs are strongly involved in the survival of *S. aureus* upon PMN treatment [31], [33], we analyzed whether there is an association between phagocytosis and the RSH-dependent stringent response. Comparison of published data revealed that the transcription pattern during neutrophil phagocytosis (Voyich *et al.*, 2005) is similar to that observed after amino acid starvation and/or mupirocin treatment [13], [20], [21], namely down-regulation of the translational machinery and de-repression of CodY regulated amino acid transporters and biosynthesis genes. Hence, we hypothesized that phagocytosis may induce an RSH-dependent stringent response, similar to the depletion of amino acids. Under these conditions, the (p)ppGpp accumulation should result in increased PSMs synthesis. Thus, an *rsh_Syn_* mutant, unable to transcribe *psms*, might be more sensitive to phagosomal killing. Indeed, the PMN bactericidal killing assay revealed that the *rsh_Syn_* mutant is significantly less able to survive after phagocytosis compared to the wild-type strains (Fig. 5A). This effect could be reversed by the introduction of full-length *rsh* into the chromosome of the *rsh_Syn_* mutants. A comparison of the two strains (HG001 and Newman) with their respective mutant derivatives revealed similar results. Also an assumed contribution of *relP* and *relQ* could be excluded, since a *relP/Q* double mutant showed no decreased survival phenotype compared to the wild-type strain (data not shown).

To further analyze whether reduced intracellular *psm* expression solely accounted for the *rsh_Syn_* defect in survival, we constructed a *psmα1-4*, *psmβ1,2* double mutant and an *agr* mutant, known to be defective in *psm* expression. The phenotype of the *psmα*, *psmβ* double mutant and the *agr* mutant closely resemble that of the *rsh_Syn_* mutant concerning intracellular survival after phagocytosis (Fig. 5B). Of note, there were no significant differences between the *psmα*, *psmβ* double and the *rsh_Syn_*, *psmα*, *psmβ* triple mutant, as well as between the *agr* single and the *rsh_Syn_*, *agr* double mutant, indicating that there are no additive effects of these mutations. We speculate that survival of phagocytosis is mediated by intracellular *psm* expression mediated by RSH dependent (p)ppGpp synthesis.

To address this assumption, we used a plasmid, in which *psmα1-4* and *psmβ1,2* were cloned behind a tetracycline inducible promoter, to induce *psmα* or *psmβ* expression in the *rsh_Syn_* mutant after phagosomal uptake. The intracellular induction of *psmα* and *psmβ* could significantly complement the reduced survival of the *rsh_Syn_* mutant (Fig. 5C). Interestingly, the survival of the *rsh_Syn_*, *agr* double mutant was not complementable, neither by *psmα* nor by *psmβ* expression.

We also performed PMN lysis assays by measuring the amount of released lactate dehydrogenase (LDH). Accordingly the mutants which are defective in *psms* expression were shown to be less toxic compared to the wild type strain (Fig. 5D).

These results indicate that the RSH-mediated stringent response in *S. aureus* plays a major role in survival PMN phagocytosis most probably through regulation of intracellular *psm* expression.

### Stringent response during phagocytosis

**Figure 6:**
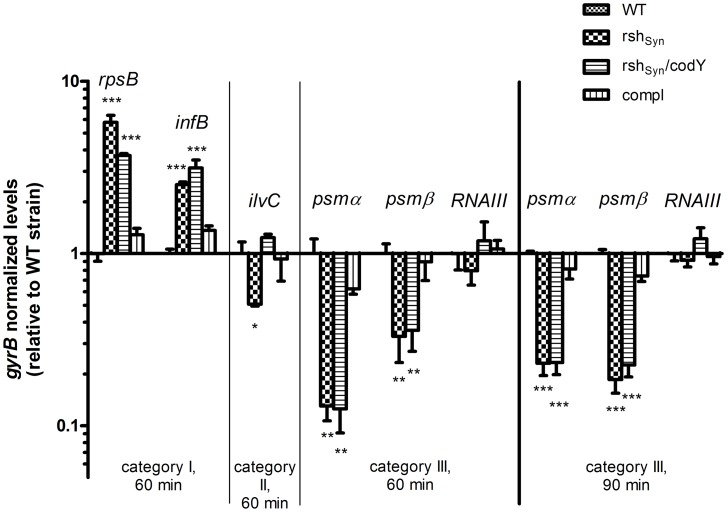
Quantitative RT-PCRs of phagocytosed bacteria. Transcript analyses of *rpsB*, *infB* (category I), *ilvC* (category II), *psmα1-4*, *psmβ1,2* (category III) and *RNAIII* genes after phagocytosis of the *S. aureus* strain HG001 and the corresponding *rsh_Syn_* mutant, *rsh_Syn_*, *codY* double mutant and the complemented *rsh_Syn_* mutant (compl). The transcripts were relatively quantified in reference of *gyrB* and are shown in a log_10_ scale relative to the wild-type strain (HG001). RNA was isolated after a 60 and 90-minute interaction of *S. aureus* with PMNs. Values from four separate experiments were used to calculate the mean expression. The levels of significance compared to the wild-type strain were determined by the two-tailed Student t test (p<0.05).

To analyze whether an RSH-dependent stringent response is induced during phagocytosis, we performed quantitative RT-PCRs from bacterial RNA obtained after 60 and 90 minutes of phagocytosis. At this stage, typically all of the bacteria are internalized [34]. We analyzed transcripts of typical genes of each of the three regulatory categories relative to the constitutive *gyrB* transcript (Fig. 6). For category I, the transcripts of *rpsB*, coding for ribosomal protein S2, and *infB*, coding for initiation factor 2 were chosen. As expected, the *rpsB* and *infB* transcription is significantly lower in the wild-type and complemented strain than in the *rsh*_Syn_ mutants (*rsh*_Syn_ single and *rsh_Syn_*, *codY* double mutant) (Fig. 6). In contrast, *ilvC* transcription (category II gene) is significantly higher in the wild-type compared to the *rsh_Syn_* mutant. Here, comparable to results of the microarray analysis, an additional *codY* mutation in the *rsh_Syn_* mutant could restore *ilvC* transcription. For category III, *psmα1-4* and *psmβ1,2* transcripts were analyzed. The wild- type and the complemented *rsh_Syn_* strain showed significantly higher transcription compared to *rsh_Syn_* mutants after incubation for 60 or 90 min with phagocytes. These results are consistent with the assumption that an RSH-mediated stringent response is induced after phagocytosis in wild-type bacteria. Of note, low expression of *psms* within the PMNs was also observed in the *rsh_Syn_*, *codY* double mutant, indicating that intracellular *psm* regulation occurs independently of CodY.

One may speculate that (p)ppGpp affect somehow the activity of the response regulator AgrA in the phagolysosomes thereby activating *psm* expression. Thus, we analyzed the intracellular expression of the prototypic AgrA target transcript *RNAIII*. However, no significant alteration of *RNAIII* transcription could be detected in the *rsh_Syn_* mutants after 60 and 90 minutes of incubation (Fig. 6). In fact, the *rsh_Syn_*, *codY* double mutant showed a slight increase of the *RNAIII* transcription compared to wild-type strain, which is in line with published data showing that *agr* transcription is slightly repressed by CodY [15], [35]. These results indicate that stringent response is induced during phagocytosis which is required for *psm* expression. The reduced transcription of *psmα1-4* in the *rsh_Syn_* mutant and *rsh_Syn_*, *codY* double mutant after phagocytosis is clearly not caused by diminished AgrA activity.

## Discussion

### A conserved “core regulon” is repressed during stringent response

To adapt to changing environmental conditions, bacteria rely on sensory and regulatory systems to modulate complex physiological processes. The stringent response is a highly conserved regulatory mechanism that is provoked by nutrient limitation. It is effective in most bacteria and is mediated by the rapid synthesis of the alarmones ppGpp or pppGpp. Previously, it was shown that, in *S. aureus*, the RSH enzyme alone is responsible for (p)ppGpp accumulation upon amino acid deprivation [13]. Here, we characterized the stringent response of *S. aureus* in a more comprehensive way. So far, no microarray studies, in which (p)ppGpp synthase mutants were compared with wild-type strains under stringent conditions, are available for *S. aureus*. For other bacteria only a few microarrays of this type were published [24], [25], [26], [36], [37]. We identified 161 genes that were significantly repressed via RSH. Most of these genes are hallmarks of the stringent response also appearing in other bacteria that have been analyzed. This conserved “core-regulon” mainly encompasses genes of the translational machinery, including initiation and elongation factors. Furthermore, genes coding enzymes of physiological processes used by dividing cells, like biosynthesis and nucleotide transport, are typically under negative stringent response. Thus, negative regulation by (p)ppGpp seems to be an evolutionarily conserved mechanism. However, the molecular mechanism by which (p)ppGpp leads to the inhibition of these genes remains mostly unclear but seems to be organism dependent [4], [5], [38], [39]. For firmicutes it was proposed that genes starting with an iGTP are repressed due to the lowering of the GTP pool [4]. We have shown previously that also in *S. aureus* stringent condition lead to lowering of the GTP level [13]. We have mapped the transcriptional start site of two rRNA operons of *S. aureus* (data not shown) and could confirm that the primary promoters initiate with iGTP which is in line with the mechanism proposed by Krasny et al., 2008 [4].

### CodY - RSH interaction

Induction of the stringent response also leads to the activation of genes proposed to be necessary for survival and maintenance. For *E. coli*, it was recently shown that most genes that are positively influenced during the stringent response are, depending on the (p)ppGpp concentration, either part of the Lrp (transcriptional regulator) or RpoS (alternative sigma factor) regulon [40]. So far, there is little indication that alternative sigma factors are involved in the stringent response in gram-positive bacteria. Instead in *S. aureus*, most of the genes that are less expressed in the *rsh_Syn_* mutant compared with the wild-type strain are part of the previously described CodY regulon [15], [16]. For these genes, introduction of a *codY* mutation into the *rsh_Syn_* mutant leads to an expression pattern that is similar to that of the wild-type strain. Thus, these genes are de-repressed upon the stringent response. An obvious link between the stringent response and CodY is the GTP pool. Amino acid deprivation leads to a lowering of the GTP pool [13], which in turn may inactivate the CodY repressor. A similar link between the stringent response and CodY was also shown for *Listeria monocytogenes* [10] and *B. subtilis* [24]. In contrast, in *Streptococci*, CodY and the stringent response seem to act independently of each other [23], [41]. This discrepancy is most likely due to species specific differences in GTP affinity of CodY [42]. In lactococci and streptococci branched-chain amino acids (e.g. isoleucine), act as the only known ligand that mediates the repressive function of CodY.

Notably, not all genes of the known CodY regulon seem to be affected under the induced stringent condition, analyzed here. Interestingly, virulence genes like the capsular gene cluster or the *agr* operon, which were found to be repressed via CodY [15], [16], did not appear to be affected during the stringent response. One explanation might be that the deactivation of the CodY repressor in the wild-type strain due to amino acid limitation is not as effective as knocking out the complete *codY* gene. Alternatively, the different CodY target genes may differ with regard to their sensitivity to CodY activity. One may assume that some of the CodY target genes are still repressed even under low GTP conditions as long as the primary CodY ligand isoleucine is present. This assumption is inline with recent findings that in *B. subtilis* CodY targets are differentially sensitive to alteration in the GTP pool: the *ilv* promoters but not the *bcaP* promoter was derepressed in a mutant not able to synthesize GTP [43].

### Regulation of PSMs

**Figure 7:**
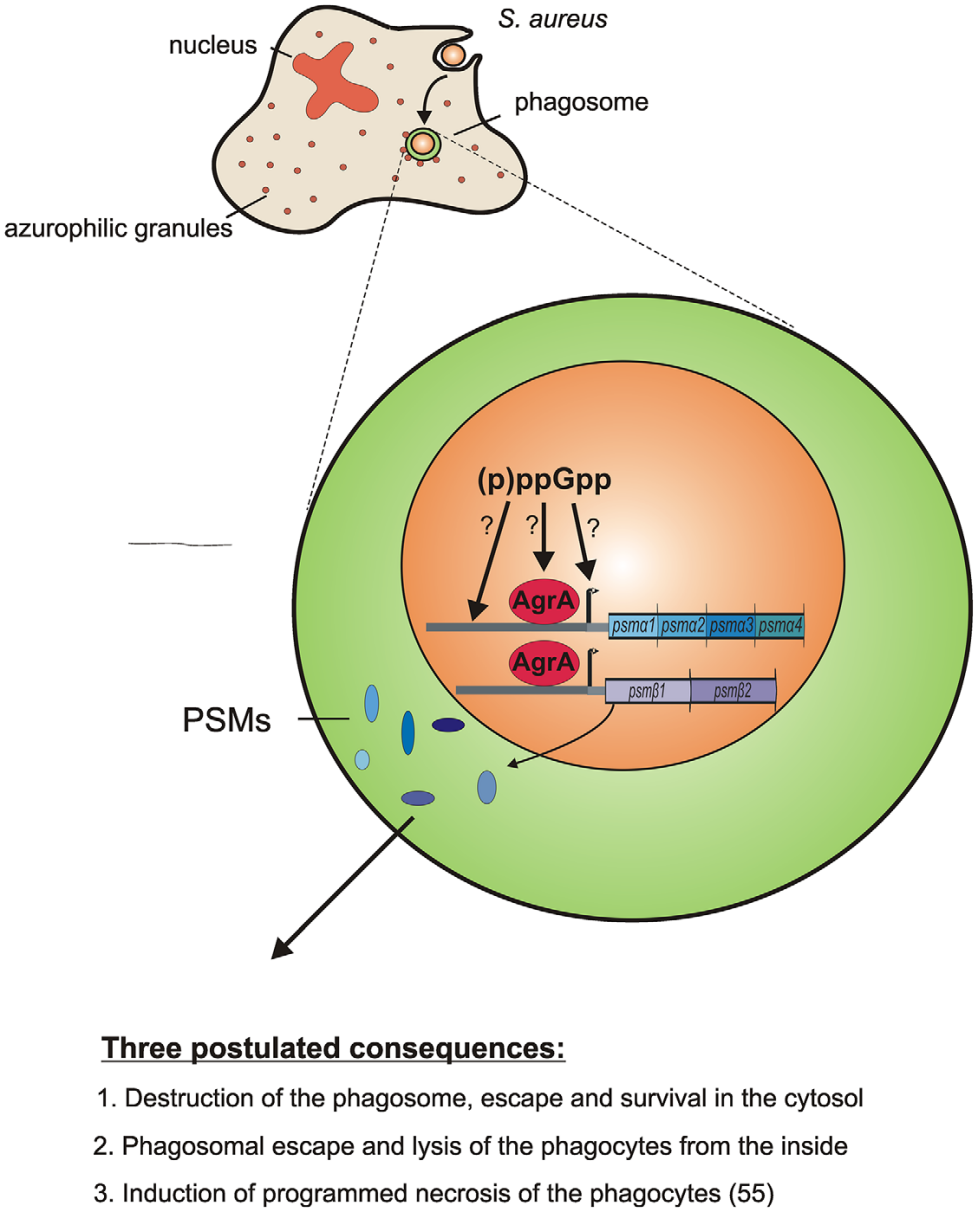
Overview of the presumed impact of the stringent response on survival after phagocytosis. The induction of the stringent response (pppGpp) in phagocytosed bacteria leads to increased *psms* expression. The PSMs in turn mediate a pronounced survival after phagocytosis, most likely through phagosomal escape or intracellular lysis of the phagocytes.

Only seven genes were positively regulated under the stringent response independently of CodY, including those coding for the cytotoxic phenol-soluble modulins (PSMs). So far *psm* transcription was shown to be directly activated via the response regulator AgrA [32]. We could show that AgrA is still essential for the stringent controlled *psmα1-4* and *psmβ1,2* transcription, since no *psms* were detectable in an *agr* mutant strain. However, the induction appears not to be due to altered *agrA* activity, since *agrA* expression was not found to be affected during the stringent response. Also after phagocytosis only *psm* transcripts but not the *Agr* effector molecule RNAIII are diminished in the *rsh_Syn_* mutants. How (p)ppGpp regulates the transcription of PSMs remains unknown. Additional regulatory elements upstream of the promoter region of *psm* genes [32] could possibly take part in the (p)ppGpp regulation, presumably mediated by unknown factor(s). In our microarray analysis, no apparent regulatory systems appeared to be affected, and thus, no candidate molecule that may indirectly be involved in (p)ppGpp dependent activation could be identified. Small molecules like (p)ppGpp or GTP may interact directly with proteins altering their enzymatic activity or binding affinities to DNA similar to the proposed GTP-CodY interaction. One may speculate that such an interaction may also alter the activity of other regulatory molecules such as the transcriptional regulator AgrA (Fig. 7).

Alternatively, it was postulated that under stringent conditions, the iNTP determines the transcription of the gene followed [4]. *psm* genes were shown to start with an iATP [32]. In *B. subtilis*, genes starting with iATP were predicted to be up-regulated after amino acid limitation through an increase of the intracellular ATP pool [4], [7]. However, this increase in ATP is obviously independent of the RSH-dependent (p)ppGpp synthesis, since we found a similar increase in the *rsh_Syn_* mutant which is in line with results obtained in *B. subtilis* (Tojo et al., 2008). Thus, increasing the ATP pool is not sufficient to trigger induction of the PSMs since no enhanced *psm* transcription was observed in the *rsh_Syn_* mutant despite a similarly elevated ATP pool than the wild-type and *rsh_Syn_* complemented strain.

### Stringent response during phagocytosis

Our results indicate, by taking the example of a gene coding for a ribosomal protein S2 (*rpsB*) and initiation factor 2 (*infB*), that the down-regulation of translational proteins after phagocytosis is mediated via *rsh* activity (Fig. 6). Together, with the lower expression of *ilvC*, *psmα1-4* and *psmβ1,2* in the *rsh_Syn_* mutants, these findings suggest that the stringent response is induced after phagocytosis (Fig. 7). This result is supported by the global transcriptional analyses of the *S. aureus* strains after uptake by professional [34] and non-professional [44] phagocytes. A transcription pattern very similar to those described, was found here especially down-regulation of typical hallmark genes of the stringent response, i.e., ribosomal proteins and up-regulation of amino acid biosynthesis genes.

However, the intracellular signal for an RSH mediated stringent response remains speculative. For *S. aureus*, so far, only amino acid limitation was shown to induce (p)ppGpp synthesis via RSH. We also analyzed other conditions such as glucose starvation as potential signals for RSH dependent (p)ppGpp synthesis but found no detectable (p)ppGpp accumulation (data not shown). Thus, these findings suggest that amino acid limitation triggers the response within phagocytes. Moreover, in *L. monocytogenes*, a gram-positive intracellular pathogen, the amino acid limitation sensing RSH was shown to be essential for survival and replication in macrophages [10]. However, this finding is in contradiction to results obtained from gram-negative intracellular pathogens. Here, the authors concluded that no amino acid limitation occurs in infected host cells. This conclusion is based on the finding that RelA, the enzyme solely responsible for accumulation of (p)ppGpp upon amino acid limitation in gram-negative bacteria, is not activated [17], [45], [46], [47], [48]. For these bacteria, the bifunctional (p)ppGpp synthase SpoT seems to be more important for adaption to the intracellular environment. It was presumed that balancing the basal levels of (p)ppGpp (due to the hydrolase and synthase activity of SpoT) is essential for intracellular adaptation [46]. There may be major differences between gram-negative and gram-positive bacteria concerning the mechanisms of signalling and responses to intracellular environments. Interestingly, treatment with azurophilic granule proteins [49] imposed transcriptional changes similar to those of the stringent response. It has to be elucidated whether parts of the reactive oxygen species (ROS) in phagolysosomes may be a trigger for the stringent response in *S. aureus*.

### PSM expression contributing to survival in phagocytes

The predominant role of *S. aureus* PSMs in killing human neutrophils was previously shown [31]. The reduced survival of the *rsh_Syn_* mutant after phagocytosis is probably due to a decreased expression of PSMs within the phagolysosome. No other candidate virulence gene which could potentially account for this observation appeared in our microarray analysis to be down-regulated in the *rsh_Syn_* mutant. Comparing the survival of an *rsh_Syn_* mutant to a *psmα*, *psmβ* double mutant or an *agr* mutant reveals a similar low survival rate which is in line with published data [33]. The distinct role of *psm*s expression for survival could be demonstrated by complementation assays of the *rsh_Syn_* mutant with tetracycline induced *psmα1-4* or *psmβ1,2*. The fact that the reduced survival of an *rsh_Syn_*, *agr* double mutant was not complementable by *psm* expression indicates that probably additional *agr* dependent factors are needed to survive after phagocytosis.

Little is known about the intracellular behaviour of *S. aureus*. Growing evidence suggests that *S. aureus* can survive after phagocytosis and persists in neutrophils or macrophages to hide from the immune system, as well as to travel to and infect distant sites in the host [50], [51], [52]. However, the importance of single virulence factors contributing to intracellular survival seems to be dependent on the type of host cell and bacterial strains analyzed. For instance, *S. aureus* can escape from the phagoendosomes of human epithelial and endothelial cells into the cytosol, which is mediated by δ-toxin, β-toxin and ß-PSMs [53]. PSMs may similarly contribute to escape from the more toxic phagolysosomes of neutrophils and thereby allow intracellular survival. Whether they also contribute to a lysis of phagocytes from the inside remains to be shown. This was recently proposed based on observations that PSMs are efficiently inhibited by human serum and therefore lyse neutrophils rather from the inside than from the outside [54]. These authors could also show that *psm* promoter activity is strongly induced after phagosomal uptake. Moreover there could be an indirect impact of PSMs on the lysis of phagocytes. Previously it could be shown that different clinical isolates of strain USA300 induced a programmed necrosis of PMNs [55]. One may assume that intracellular expressed PSMs thereby could play an important role for this induction.

The results we obtained are significantly important for the question how and where PSMs can act as potent cytolytic molecules The data of the current study support the conclusion that in phagolysosomes a stringent response is activated and that the activity of the (p)ppGpp synthase RSH is essential for the intracellular induction of *psmα1-4 and psmβ1,2* expression. These cytolytic peptides in turn are responsible for the ability of *S. aureus* to survive after phagocytosis (Fig. 7). The signalling mechanisms leading to the activation of the (p)ppGpp synthases as well as the mechanism leading to gene activation through (p)ppGpp after phagocytosis is largely unknown and needs further examination.

## Materials and Methods

### Strains and growth conditions

Strains and plasmids are listed in Table S1. *S. aureus* strains were grown in CYPG (10 g/l casamino acids, 10 g/l yeast extract, 5 g/l NaCl, 0.5% glucose and 0.06 M phosphoglycerate) [56] or in a chemically defined medium (CDM) [15]. For strains carrying tetracycline, erythromycin or chloramphenicol resistance genes, antibiotics were used only in precultures at concentration of 5 µg/ml for tetracycline and 10 µg/ml for erythromycin and chloramphenicol, respectively. Bacteria from an overnight culture were diluted to an initial optical density at OD_600_ of 0.05 in 25 ml fresh medium using 100 ml baffled flasks and grown with shaking (220 rpm) at 37°C to the desired growth phase. For down-shift experiments, strains were grown in complete CDM including leucine/valine (leu/val) to an OD_600_ of 0.5. The cultures were filtered over a 0.22 µm filter applying vacuum, washed twice with sterile phosphate buffered saline (PBS) and bacteria were re-suspended in an equal volume of CDM medium with or without leu/val and grown for another 30 minutes. For serine starvation experiments, bacteria were re-suspended in an equal volume of CDM medium containing serine hydroxamate (SHX, 1.5 mg/ml) and incubated for 30 minutes.

### Determination of haemolytic activities on sheep blood agar plates

The haemolysis test was performed as described previously [29]. Briefly, bacteria to be tested are streaked at a right angle to RN4220 and the plate was incubated overnight. ß-hemolysin of strain RN4220 and *δ*-hemolysin of strains to be tested form a zone of clear haemolysis (synergistic effect) on blood agar plates.

### Construction of mutant strains and complementation

The *rsh_Syn_*, *codY* double mutant was obtained by transducing the *codY::tet(K)* mutation into *S. aureus* strain Newman *rsh_Syn_* [13] using Φ11 lysates of strains RN4220-21 [15]. Transductants were verified by PCR and PFGE. For complementation the full length *rsh* gene with a 960 bp upstream region was transduced into strain Newman *rsh_Syn_* (Table S1) using Φ11 lysates of strain CYL316-199 [13].

The *psmα* and *psmβ* mutants were obtained by replacing *psmα1-4* with a tetracycline resistance cassette and *psmβ1-2* with an erythromycin resistance cassette using a newly developed temperature-sensitive shuttle vector pBASE6. This vector is based on the previously described pBT2 vector [57] with the additional advantage of counter-selection against the plasmid by inducible expression of *S. aureus secY* antisense RNA of the pKOR1 vector [58]. Therefore the HindIII-Bst1107I fragment of pBT2 was replaced by the 4.875 kb EcoRV-HindIII (partial digest) fragment of pKOR1, containing the *tetR*/*secY* regulatory unit. Since some of the restriction sites of the pBT2 multiple cloning site (MCS) are also present in the introduced pKOR1 part, the MCS was removed by EcoRI-HindIII digestion. Primers: mcsmod1 (ATTCCGGAGCTCGGTACCCGGGCTAGCGCGCAGATCTGTCGACGATATCA) and mcsmod2 (AGCTTGATATCGTCGACAGATCTGCGCGCTAGCCCGGGTACCGAGCTCCGG) were mixed in equimolar amounts, heated to 95°C and slowly cooled down to room temperature. This new MCS contains only unique restriction sites and was ligated into the EcoRI-HindIII digested vector, resulting in pBASE6.

For gene replacements, two fragments flanking the *pmsα1-4*, *psmβ1-2* locus, the tetracycline and erythromycin resistant cassette were amplified and annealed by overlapping PCR to generate the *pmsα1-4-tetM* and *psmβ1,2-ermC* mutagenesis vectors pCG307 and pCG308, respectively. The amplicons were cloned into the *BglII/SalI* restriction sites of pBASE6. These plasmids were used to mutagenize strain RN4220 as described previously [13]. The obtained *psm* gene replacement mutant strains (RN4220-307 and RN4220-308) were verified by PCR. In the mutants the whole *psmα1-4* operon respectively *psmβ1,2* operon was replaced by the corresponding resistant cassette. The *rsh_Syn_*, *psmα*, *psmβ* triple mutant was obtained by transducing the *psmβ::erm(C)* and *psmα::tet(M)* mutations into *S. aureus* strain *HG001 rsh_Syn_* using lysates of strain RN4220-307 and RN4220-308.

### RNA isolation

RNA isolation for microarray analysis and northern blot analysis was performed as described previously [59]. Briefly, bacteria were lysed in 1 ml of Trizol reagent (Invitrogen Life Technologies, Karlsruhe, Germany) with 0.5 ml zirconia-silica beads (0.1 mm-diameter) in a high-speed homogenizer (Savant Instruments, Farmingdale, NY). RNA was isolated as described in the instructions provided by the manufacturer of Trizol.

RNA isolation after phagocytosis was performed as described in the instructions provided by the manufacturer of the RNA isolation kit (ExpressArt RNAready, AmpTec) with the modification adding an inhibitor removal buffer (high pure viral nucleic acid kit, Roche diagnostics) in the first step. Then DNA digestion was performed as instructed by the RNA isolation kit.

### Northern blot analysis

Northern blot analyses were performed as described previously [59]. Digoxigenin-labeled probes for the detection of specific transcripts were generated using a DIG-Labeling PCR Kit following the manufacturer's instructions (Roche Biochemicals). Oligonucleotides were used for probe generation as described previously [13], [15], [60] or are listed in Table S2.

### Reverse transcription and quantitative real-time PCR

Relative quantifications of *α-type psms*, *β-type psms*, *infB*, *rpsB*, *RNAIII* and *gyrB* transcripts were performed using LightCycler instrument (Roche). Briefly, RNA isolated from cultures after phagocytosis (60 and 90 min) was transcribed into complementary DNA using SuperScriptIII Reverse Transcriptase (Invitrogen) and 200 ng of random hexamer primers (Fermentas). Complementary DNA was diluted 1∶5 and quantitative real-time PCR was performed using the QuantiFast SYBR Green PCR Kit (Qiagen). A standard curve for each gene was generated using 5-fold serial dilutions of wild-type HG001 cDNA at timepoint 0 h (oligonucleotides see Table S2). Statistical analysis was performed with the Prism software package (version 5.0; GraphPad) using the Student *t* test two-tailed analysis (p<0.05).

### Quantitative measurement of the intracellular ATP pool

*S. aureus* wild type HG001, isogenic mutants and the complemented strain were grown in CDM to OD_600_ = 0.5. Cells were shifted to CDM with and without leu/val as described above. Samples for intracellular ATP analysis were harvested 30 min after the shift by fast filtration over a 0.22 µm sterility filter applying vacuum. Cells were washed, quenched and nucleotides were extracted as described recently [61], [62]. The extracts were resuspended in 5 ml of 0.1 M Tris-acetate buffer (pH 7.75) and stored at −80°C. The detection of ATP was performed by the Enlighten ATP assay system using luciferase and luciferin (Promega). Therefore nucleotide extracts were diluted 1∶100 in Tris-acetate buffer and 10 µl mixed with 90 µl of the ATP assay reagents. Luminescence was measured with a luminescence detection reader (Infinite M200Pro, Tecan, Austria). The standard curve was generated by using known concentrations of ATP. The results of the intracellular ATP measurements represent the mean of 2 biological replicates measured in triplicates. Statistical analysis was performed with the Prism software package (version 5.0; GraphPad) using the Student *t* test two-tailed analysis (p<0.05).

### Microarray manufacturing and microarray design

The microarray was manufactured by in situ synthesis of 60-base-long oligonucleotide probes (Agilent, Palo Alto, CA), selected as previously described [63]. The array covers >98% of all open reading frames (ORFs) annotated in strains N315 and Mu50, MW2, COL, NCTC8325 and USA300, and MRSA252 and MSSA476, as well as Newman, including their respective plasmids.

### Preparation of labeled nucleic acids for expression microarrays

Total RNA was purified from strain HG001 WT, *rsh_Syn_* mutant, *codY* mutant and *rsh_Syn_*, *codY* double mutant grown in CDM to an OD_600_ of 0.5. For each strain RNA of three independently grown cultures was analyzed. After additional DNase treatment, the absence of remaining DNA traces was confirmed by quantitative PCR (SDS 7700; Applied Biosystems, Framingham, MA) with assays specific for 16S rRNA [15]. Batches of 5 µg of total *S. aureus* RNA were labeled by Cy3-dCTP using SuperScript II (Invitrogen, Basel, Switzerland) following the manufacturer's instructions. Labeled products were then purified onto QiaQuick columns (Qiagen). Purified genomic DNA from the different sequenced strains used for the design of the microarray was extracted (DNeasy; Qiagen), labeled with Cy5 dCTP using the Klenow fragment of DNA polymerase I (BioPrime, Invitrogen, Carlsbad, CA), and used for the normalization process [64] Cy5-labeled DNA (500 ng) and a Cy3-labeled cDNA mixture were diluted in 50 µl of Agilent hybridization buffer and hybridized at a temperature of 60°C for 17 h in a dedicated hybridization oven (Robbins Scientific, Sunnyvale, CA). Slides were washed, dried under nitrogen flow, and scanned (Agilent, Palo Alto, CA) using 100% photon multiplier tube power for both wavelengths.

### Microarray analysis

Fluorescence intensities were extracted using Feature Extraction software (version 9; Agilent). Local background-subtracted signals were corrected for unequal dye incorporation or unequal load of the labeled product. The algorithm consisted of a rank consistency filter and a curve fit using the default LOWESS (locally weighted linear regression) method. Data consisting of three independent biological experiments were expressed as log 10 ratios and analyzed using GeneSpring, version 8.0 (Silicon Genetics, Redwood City, CA). A filter was applied to select oligonucleotides mapping ORFs in the Newman genome, yielding approximately 95% coverage. Statistical significance of differentially expressed genes was calculated by analysis of variance [65] using GeneSpring, including the Benjamini and Hochberg false discovery rate correction of 5% (*p* value cutoff, 0.05) and an arbitrary cutoff of twofold for expression ratios.

### Microarray data accession number

The complete microarray data set has been posted on the Gene Expression Omnibus database (http://www.ncbi.nlm.nih.gov/geo/) under accession number GSE99340 for the platform design and GPL7137 for the original data set.

### PMN bactericidal activity

Killing by human neutrophils was performed as described previously [66]. Briefly, bacteria of the logarithmic phase were washed and adjusted in potassium phosphate buffer (10 mM K_2_PO_4_). Neutrophils were isolated from peripheral blood of healthy volunteers by ficoll/histopaque gradient centrifugation as described previously [67] and resuspended in HBSS-HSA (hank's balanced salt solution, Sigma, containing 0.05% human serum albumin). Bacteria were opsonized by addition of pooled human serum (Sigma) to a final concentration of 10%. Opsonized bacteria (10^7^/ml) and neutrophils (10^6^/ml) were combined to a volume of 500 µl and samples were shaken at 37°C. For the *psm* complementation assays, neutrophils and bacteria were preincubated for 30 minutes without anhydrotetracycline (ATc) followed by addition of 0,1 µg/ml ACT for another 30 minutes. Aliquots were diluted in ice-cold water and vortexed vigorously to disrupt the neutrophils and halt bacterial killing. Appropriate dilutions were plated on tryptic soy agar plates and incubated at 37°C for the following day for enumeration of CFU. The percent of bacterial survival was calculated with the equation CFU_+PMN_ at t_60_/CFU_+PMN_ at t_0_. Statistical analysis was performed with the Prism software package (version 5.0; GraphPad) using the Student *t* test two-tailed analysis (p<0.05).

### *S. aureus*-induced PMN lysis

Following phagocytosis of *S. aureus*, lysis of human neutrophils was determined with a standard assay for release of lactate dehydrogenase (LDH) as described by the manufacturer (Cytotoxicity Detection kit; Roche Applied Sciences). Statistics were performed with the Prism software package (version 5.0; GraphPad) using the Student *t* test two-tailed analysis (p<0.05).

## Supporting Information

Table S1 Strains and plasmids used in the study.
(DOC)

Table S2 Oligonucleotides used for the construction of hybridisation probes and for qt-RT PCR.
(DOC)
