# Supplementary material for: The Stringent Response of Staphylococcus aureus and Its Impact on Survival after Phagocytosis through the Induction of Intracellular PSMs Expression
Source: PLoS Pathog. 2012 Nov 29;8(11):e1003016. doi: 10.1371/journal.ppat.1003016 (PMC3510239; doi:10.1371/journal.ppat.1003016)
Supplement: Table S1 — Strains and plasmids used in the study. (DOC) [file ppat.1003016.s001.doc]

**Table S1: Strains and plasmids**

| **Strain** | **Description** | **Reference** |
| --- | --- | --- |
| RN4220-21 | RN4220 *codY*::*tet*(K) |  |
| CYL316-199 | RN4220, pYL112Δ19, L54 *int* gene, r^-^ with pCG199 for *rsh* complementation |  |
| HG001 | *rsbU* restored RN1 (8325), previously named RN1HG |  |
| HG001-86 | HG001 *rsh_Syn_* (Δ942-950nt) |  |
| HG001-21 | HG001 *codY*::*tet*(K) |  |
| HG001-86-21 | HG001 *rsh_Syn_* (Δ942-950nt), *codY*::*tet*(K) mutant |  |
| HG001-86-199 | HG001 *rsh_Syn_* (Δ942-950nt) complementation with full length *rsh* |  |
| HG001-agr | HG001 *agr::tet*(M) | This work |
| HG001-86-agr | HG001 *rsh_Syn_* (Δ942-950nt), *agr::tet*(M) | This work |
| HG001-307-308 | HG001 *psmα1-4::tetM, psmβ1-2::ermC* mutant | This work |
| HG001-86-307-308 | HG001 *rsh_Syn_* (Δ942-950nt), *psmα1-4::tetM, psmβ1-2::ermC* mutant | This work |
| HG001-86-psmα | HG001 *rsh_Syn_* (Δ942-950nt) complementation with tetracycline inducible *psmα1-4* | This work |
| HG001-86-psmβ | HG001 *rsh_Syn_* (Δ942-950nt), complementation with tetracycline inducible *psmβ1-2* | This work |
| HG001-86-agr-psmα | HG001 *rsh_Syn_* (Δ942-950nt), *agr::tet*(M) complementation with tetracycline inducible *psmα1-4* | This work |
| HG001-86-agr-psmβ | HG001 *rsh_Syn_* (Δ942-950nt), *agr::tet*(M) complementation with tetracycline inducible *psmβ1-2* | This work |
| Newman | Wild-type |  |
| Newman-86 | Newman *rsh_Syn_* (Δ942-950nt) |  |
| Newman-21 | Newman *codY::tet(K)* |  |
| Newman-86-21 | Newman *rsh_Syn_* (Δ942-950nt), *codY::tet(K)* mutant | This work |
| Newman-86-199 | Newman *rsh_Syn_* (Δ942-950nt) complementation with full-length *rsh* | This work |
| Newman-agr | Newman *agr::tet(M)* |  |
| RN6911 | *agr::tet(M)* |  |
| **Plasmid** | **Description** | **Reference** |
| pPSMα-Cerulean | *psmα1-4* cloned behind a tetracycline inducible promoter |  |
| pPSMβ-Cerulean | *psmβ1-2* cloned behind a tetracycline inducible promoter |  |
| pCG75 | pCR2.1 vector (Invitrogen) with a *tet(M)* resistant cassette of strain RN 6911 | This work |
| pMUTIN4 | integrative vector including the IPTG-inducible promoter Pspac, (Ap^r^, Em^r^) |  |
| pBASE6 | tetracycline inducible suicide mutagenesis vector | This work |
| pCG307 | pBASE6 vector with *psmα1-4::tet(M)* replacement | This work |
| pCG308 | pBASE6 vector with *psmβ1-2::erm(C)* replacement | This work |

**Table S1: References**

1. Pohl K, Francois P, Stenz L, Schlink F, Geiger T, et al. (2009) CodY in *Staphylococcus aureus*: a regulatory link between metabolism and virulence gene expression. J Bacteriol 191: 2953-2963.

2. Geiger T, Goerke C, Fritz M, Schafer T, Ohlsen K, et al. (2010) Role of the (p)ppGpp synthase RSH, a RelA/SpoT homolog, in stringent response and virulence of Staphylococcus aureus. Infect Immun 78: 1873-1883.

3. Duthie ES, Lorenz LL (1952) Staphylococcal coagulase; mode of action and antigenicity. J Gen Microbiol 6: 95-107.

4. Wolz C, McDevitt D, Foster TJ, Cheung AL (1996) Influence of *agr* on fibrinogen binding in *Staphylococcus aureus* Newman. Infect Immun 64: 3142-3147.

5. Novick RP, Ross HF, Projan SJ, Kornblum J, Kreiswirth B, et al. (1993) Synthesis of staphylococcal virulence factors is controlled by a regulatory RNA molecule. EMBO J 12: 3967-3975.

6. Giese B, Glowinski F, Paprotka K, Dittmann S, Steiner T, et al. (2011) Expression of delta-toxin by Staphylococcus aureus mediates escape from phago-endosomes of human epithelial and endothelial cells in the presence of beta-toxin. Cell Microbiol 13: 316-329.

7. Vagner V, Dervyn E, Ehrlich SD (1998) A vector for systematic gene inactivation in *Bacillus subtilis*. Microbiology 144 ( Pt 11): 3097-3104.
