# Supplementary material for: The Stringent Response of Staphylococcus aureus and Its Impact on Survival after Phagocytosis through the Induction of Intracellular PSMs Expression
Source: PLoS Pathog. 2012 Nov 29;8(11):e1003016. doi: 10.1371/journal.ppat.1003016 (PMC3510239; doi:10.1371/journal.ppat.1003016)
Supplement: Table S2 — Oligonucleotides used for the construction of hybridisation probes and for qt-RT PCR. (DOC) [file ppat.1003016.s002.doc]

**Table S2: Oligonucleotides used for the construction of hybridisation probes and for qt-RT PCR**

| **Description** | **Template** | **Name^*^** | **Sequence** |
| --- | --- | --- | --- |
| *agrA* | HG001  HG001 | agrADIG-U  agrADIG-L | ATGATAGAAGAAAAGCCTATGG  CTAAATGGGCAATGAGTCTG |
| *psm (α1-4)* | HG001  HG001 | psmαDIG-U  psmαDIG-L | CATCGTTTTGTCCTCCTG  TCATCGCTGGCATCATTA |
| *psm (β1,2)* | HG001  HG001 | psmβDIG-U  psmβDIG-L | TAACGCAATTAAAGATACCG  TCATGTTGTTGTGCAGCTTG |
| *brnQ1* | HG001  HG001 | brnQ1DIG-U  brnQ1DIG-L | GTAAAGCCCAACCAACAGGT  TCATCGTAGGTTTAACAGCA |
| *rpsB* |  |  |  |
| *infB* |  |  |  |
| *Tsf* |  |  |  |
| *guaC* |  |  |  |
| *ilvC* |  |  |  |
| *rpsB-qt RT PCR* | HG001  HG001 | rpsBqtRT-U  rpsBqtRT-L | ATCTCAAAACGAATCAAACG  GGTCAACTACGAATAATGCTT |
| *RNAIII-qt RT PCR* | HG001  HG001 | rnaIIIqtRT-U  rnaIIIqtRT-L | CGACACAGTGAACAAATTC  CGATGTTGTTTACGATAGC |
| *infB-qt RT PCR* | HG001  HG001 | infBqtRT-U  infBqtRT-L | ATAAACCACAAAATCAACCAGC  TCAACGCCATAATCATCG |
| *ilvC-qt RT PCR* | HG001  HG001 | ilvCqtRT-U  ilvCqtRT-L | TGAAACATTAGTAGAAGCGG  CGTGGTCCTGAAACATAGTC |

U: upper primer, L: lower primer
